# Six-Month Chronic Toxicity Study of Tamarind Pulp (*Tamarindus indica* L.) Water Extract

**DOI:** 10.3390/scipharm85010010

**Published:** 2017-03-09

**Authors:** Irene Iskandar, Finna Setiawan, Lucy D N Sasongko, I Ketut Adnyana

**Affiliations:** 1 Pharmacology and Clinical Pharmacy Research Group, School of Pharmacy, Institut Teknologi Bandung, Bandung 40132, Indonesia; irene.iskandar41@gmail.com (I.I.); finna_florentina@yahoo.com (F.S.); 2 Faculty of Pharmacy, University of Surabaya, Surabaya 60293, Indonesia; finna_florentina@yahoo.com; 3 Pharmaceutics Research Group, School of Pharmacy, Institut Teknologi Bandung, Bandung 40132, Indonesia; lucys@fa.itb.ac.id

**Keywords:** *Tamarindus indica*, tamarind, toxicity

## Abstract

Tamarind water extract has been shown to demonstrate an anti-obesity effect. In this research, long-term use of tamarind pulp water extract safety was evaluated. Tamarind pulp was extracted by reflux method, followed by freeze-drying to obtain dry extract. Wistar rats were divided into six groups, with 20 animals of each sex per group. The control group and satellite control group received carboxymethylcellulose sodium (CMC-Na) 0.5% 1 mL/100 g bw (body weight) per day. Treatment groups received tamarind pulp extract at doses of 75, 200, 1000, satellite 1000 mg/kg bw per day for six months. After six months, control groups and the treatment group were sacrificed. Satellite groups were sacrificed one month later. Relative organ weights, hematology and clinical biochemistry profiles were determined. After six months, there were no significant change in body weight, hematologic, and clinical biochemistry profiles of the tested group. Body weight of male rats in the satellite 1000 mg/kg bw group was significantly increased in week 30 compared to the satellite control group (*p* < 0.05). The relative spleen weight of female rats of the 200 mg/kg bw group was reduced (*p* < 0.05). The relative kidney weight of male rats in the 1000 mg/kg bw group was increased (*p* < 0.05). This study showed that tamarind pulp extract was generally safe and well tolerated at the tested dose.

## 1. Introduction

*Tamarindus indica* L. is a tropical evergreen tree native to the fertile area throughout Africa and Southern Asia [1]. Tamarind pulp water extract contains sterol, terpene, saponin, citric acid, tartaric acid, and malic acid [2]. Tamarind pulp water extract has an anti-obesity effect in high fat–induced rats or high carbohydrate–induced rats. Tamarind pulp water extract decreases total cholesterol, low density lipoprotein (LDL), and triglyceride levels [3]. Tamarind pulp contains a considerable amount of polyphenols and flavonoids and shows antioxidant activity [4]. Another study showed that tamarind leaves’ fluid extract has an antioxidant property. Acute oral toxicity tests showed that tamarind leaves’ fluid extract is a non-toxic substance. However, oral mucous irritability tests showed that tamarind leaves’ fluid extract is a milid irritant due to several organic acids such as tartaric, malic and citric acids [5]. To use tamarind pulp water extract as an anti-obesity traditional medicine, the safety of tamarind pulp water extract should be evaluated. In a previous study, the acute and sub-chronic toxicity of tamarind pulp water extract was investigated. The acute toxicity study of tamarind pulp extract showed there was no mortality after the administration of 5000 mg/kg bw (body weight) and 3000 mg/kg bw tamarind pulp extract [4]. The LD50 (lethal dose 50) of tamarind pulp extract is greater than 5000 mg/kg bw and can be classified as practically non-toxic and is considered safe by the recommendation of the World Health Organization (WHO) and the Organization of Economic and Cultural Development (OECD) [6]. The sub-acute toxicity study of tamarind pulp water extract showed that there was no mortality after 28 days of administration of tamarind pulp water extract [7]. However, the safety of long-term administration of tamarind pulp water extract has not been investigated. The aim of this study was to evaluate the long-term safety of tamarind pulp water extract as an anti-obesity traditional medicine. In this research, tamarind pulp water extract was administered to rats, and after six months of administration of tamarind pulp water extract, toxicity parameters such as clinical biochemistry and hematology profiles, body weight changes and relative organ weights of rats were determined.

## 2. Materials and Methods

### 2.1. Plant Material Collection

Fresh tamarind pulp was collected from Tamarind tree grows in Cijati village, Majalengka, West Java, Indonesia. Tamarind pulp were identified and characterized in Herbarium Bandungense, School of Life Science and Technology, Institut Teknologi Bandung.

### 2.2. Tamarind Pulp Water Extract Preparation

Tamarind pulp was soaked in distilled water with tamarind pulp to water ratio of 1:10. The mixture was boiled in reflux apparatus for 150 min after water boiled, and then filtered with filter paper. Filtrate was dried with freeze dryer (Telstar LyoQuest, Telstar, Terrassa, Spain) to gain dry extract.

### 2.3. Experimental Animal

One-hundred and twenty male and female normal Wistar rats, six to eight weeks old, weighing 150–200 g were acclimatized in room temperature, with 12 h light and 12 h dark cycle, with free access to food and drink for one to two weeks. The animals were randomly divided into six groups, 20 animals for each sex per group.

Group 1: Control group received carboxymethylcellulose sodium (CMC-Na) 0.5% 1 mL/100 g bw per day for six months.
Group 2: Control satellite group received CMC-Na 0.5% 1 mL/100 g bw per day for six months.
Group 3: 75 mg/kg bw group received tamarind pulp extract 75 mg/kg bw per day for six months.
Group 4: 200 mg/kg bw group received tamarind pulp extract 200 mg/kg bw per day for six months.
Group 5: 1000 mg/kg bw group received tamarind pulp 1000 mg/kg bw per day for six months.
Group 6: Satellite 1000 mg/kg bw group received tamarind pulp 1000 mg/kg bw per day for six months.

After six months of administration of tamarind pulp water extract, control group, 75 mg/kg bw group, 200 mg/kg bw group and 1000 mg/kg bw group were sacrified immediately. Control satellite group and satellite 1000 mg/kg bw were monitored for a month and then were sacrificed.

### 2.4. Six-Month Chronic Toxicity Study

Tamarind pulp water extract suspension was administered orally to the test group six days per week for six months. Control and satellite control group received CMC-Na 0.5% solution, 1 mL/100 g bw. After six months, control groups, and treatment group were sacrificed. Satellite groups (Group 2 and Group 6) were sacrificed one month later. Relative organ weights, hematologic and clinical biochemistry profiles, were determined.

### 2.5. Relative Organ Weights Determination

At the end of the study, the rats were weighed and sacrificed using carbon dioxide chamber. Livers, hearts, spleens, lungs, adrenals, ovaries, uterus, testes, seminal vesicles, and kidneys were removed from the rats and weighed. Relative organ weights were determined by calculating ratio between organ weight and body weight.

### 2.6. Hematological Analysis

Determination of hematocrit, white blood cell counts, red blood cell counts, platelet counts, haemoglobin, mean corpuscular volume (MCV), mean corpuscular hemoglobin (MCH), and mean corpuscular hemoglobin concentration (MCHC) were determined by hematology analyzer (Medonic M-Series, Medonic, Stockholm, Sweden). The measurement is based on impedance and spectrophotometry principle.

### 2.7. Biochemistry Profile Analysis

#### 2.7.1. Blood Glucose Determination

Blood glucose determination was performed by enzymatic colorimetric test according to the method of Barham and Trinder. The glucose was determined after enzymatic oxidation in the presence of glucose oxidase. The formed hydrogen peroxidase reacts under catalysis of peroxidase with phenol and 4-aminophenazone to a red-violet quinoneimine dye as indicator [8]. The absorbance of sample and reagent was measured by spectrophotometer (Microlab 300, ELITechGroup, Dieren, Netherlands) at 546 nm after 10 min incubation.

#### 2.7.2. Total Cholesterol Determination

Total cholesterol determination was performed by enzymatic colorimetric test, CHOD-PAP (Cholesterol oxidase/p-aminophenazone) method. Cholesterol was determined after enzymatic hydrolysis and oxidation. The indicator quinoneimine is formed from hydrogen peroxidase and 4-aminophenazone in the presence of phenol and peroxidase [9]. The absorbance of sample and reagent was measured by spectrophotometer (Microlab 300, ELITechGroup, Dieren, Netherlands) at 546 nm after 10 min incubation.

#### 2.7.3. Triglyceride Determination

Triglyceride was determined by enzymatic colorimetric test, GPO-PAP (Glycerol-3-Phosphate oxidase/p-aminophenazone) method. Triglycerides were determined after enzymatic hydrolysis with lipases. Indicator quinoneimine was formed from hydrogen peroxidase, 4-aminoantipyrine and 4-chlorophenol under the catalytic influence of peroxidase [10]. The absorbance of reagent and sample was measured by spectrophotometer (Microlab 300, ELITechGroup, Dieren, Netherlands) at 546 nm after 10 min incubation.

#### 2.7.4. Aspartate Amino Transferase Determination

Aspartate Amino Transferase (AST) was determined by kinetic method for determination of AST activity according to recommendation of the Expert Panel of the International Federation of Clinical Chemistry (IFCC) [11]. Absorbance was measured with spectrophotometer (Microlab 300, ELITechGroup, Dieren, Netherlands) at 365 nm, after 1, 2 and 3 min after sample and reagent mixed and incubated.

#### 2.7.5. Alanine Amino Transferase Determination

Alanine Amino Transferase (ALT) was determined by kinetic method for determination of ALT activity according to recommendation of the Expert Panel of the IFCC [12]. Absorbance was measured with spectrophotometer (Microlab 300, ELITechGroup, Dieren, Netherlands) at 365 nm, after 1, 2 and 3 min after sample and reagent mixed and incubated.

#### 2.7.6. Blood Creatinine Determination

Creatinine was determined by Jaffe reaction, using photometric colorimetric test for kinetic determination of creatinine at 25 °C and 37 °C without deproteinization. Creatinine forms in alkaline solution as an orange-red colored complex with picric acid. The absorbance of this complex is proportional to the creatinine concentration in the sample [13]. The absorbance of sample and reagent was measured with spectrophotometer (Microlab 300, ELITechGroup, Dieren, Netherlands) at 492 nm, 30 min and 2 min after sample and reagent were mixed.

#### 2.7.7. Urea

Blood urea was determined by Berthelot method using enzymatic colorimetric test. Urea is hydrolyzed in the presence of water and urease to produce ammonia and carbon dioxide. In a modified Berthelot reaction the ammonium ions react with hypochlorite and salicylate to form a green dye. The absorbance increase at 578 nm is proportional to the urea concentration in the sample [14,15].

### 2.8. Statistics

In this study, data were expressed as mean ± standard deviation (SD). Data were analyzed, the significance of the difference between mean was established by one-way analysis of variance (ANOVA) with *p* < 0.05 considered significant.

## 3. Results

### 3.1. Effect of Tamarind Pulp Water Extract on Body Weight Profile

**Figure 1:**
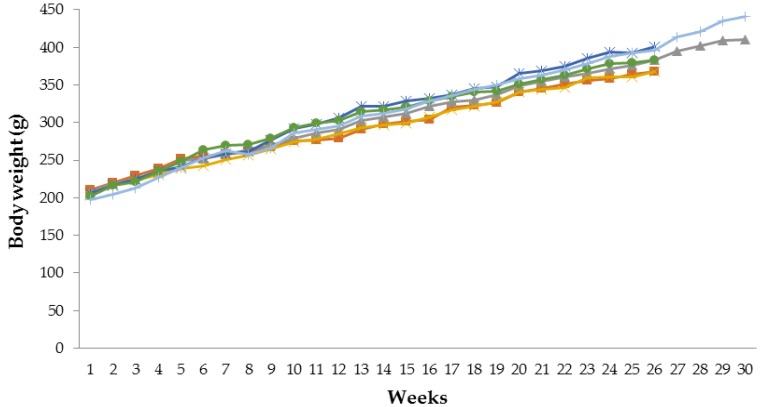
Body weight profiles of male rats treated with tamarind pulp extract for six months. (■) Control group; (▲) satellite control group; (×) 75 mg/kg bw group; (*) 200 mg/kg bw group; (●) 1000 mg/kg bw group; (|) satellite 1000 mg/kg bw group.

**Figure 2:**
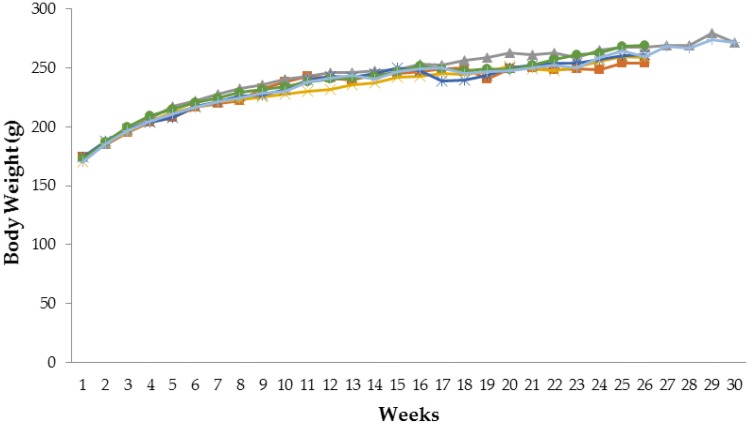
Body weight profiles of female rats with Tamarind pulp extract for six months. (■) control group; (▲) satellite control group; (×) 75 mg/kg bw group; (*) 200 mg/kg bw group; (●) 1000 mg/kg bw group; (|) satellite 1000 mg/kg bw group.

Figure 1 and Figure 2 show the body weight profiles of male and female rats for six months of tamarind pulp extract administration. There was no significant difference in the body weight profile of the test group compared to the control group (*p* > 0.05). However, the body weights of the male rats in the satellite 1000 mg/kg bw group were significantly increased (*p* < 0.05) in week 30 compared to the satellite control group (440.70 ± 38.90 g vs. 410.25 ± 54.68 g).

### 3.2. Effect of Tamarind Pulp Water Extract on Hematology Profile

Table 1 and Table 2 show the hematology profiles of male and female rats, after six months of administration of tamarind pulp water extract. There were no significant differences (*p* > 0.05) between the test group and control group in hematological parameters such as white blood cell counts, red blood cell counts, hemoglobin, MCH, MCHC, MCV, hematocrit, and platelet counts.

### 3.3. Effect of Tamarind Pulp Water Extract on Relative Organ Weights

Table 3 and Table 4 show the relative organ weights of male and female rats after six months of administration of tamarind pulp water extract. There were no significant differences in the relative liver weight, relative lung weight, relative heart weight, relative spleen weight, relative adrenal weight, relative seminal vesicle weight and relative testicle weight of male rats compared to the control group. However, there were significant increases (*p* < 0.05) in the relative kidney weights of male rats in the 1000 mg/kg bw group compared to the control group (0.70 ± 0.07 vs 0.63 ± 0.05). There were no significant differences in the relative liver weight, relative lung weight, relative heart weight, relative kidney weight, relative adrenal weight, relative uterus weight, and relative ovaries weight of female rats compared to the control group. The relative spleen weight of female rats of the 200 mg/kg bw group was reduced (*p* < 0.05).

### 3.4. Effect of Tamarind Pulp Water Extract on Clinical Biochemistry Profile

Table 5 and Table 6 show the clinical biochemistry profile of male and female rats after six months of administration of tamarind pulp water extract. There was no significant difference in clinical biochemistry parameters such as glucose, cholesterol, triglyceride, AST, ALT, urea, and blood creatinine of male rats and female rats compared to the control group.

## 4. Discussion

Tamarind pulp water extract has an anti-obesity effect in high fat–induced rats [3] and high carbohydrate–induced rats [16]. Research showed that tamarind pulp water extract decreased LDL levels and triglyceride levels [3]. Finna et al. showed that tamarind pulp water extract has an anti-obesity effect in a high carbohydrate–induced diet at the dose of 75 mg/kg bw [16]. To use tamarind pulp water extract as an anti-obesity traditional medicine, the safety of tamarind pulp water extract should be determined. The dose selected was started at 75 mg/kg bw. In a chronic toxicity study, the test substance is administered for a long enough time for a chronic effect to be realized [17]. Satellite groups were included to monitor the reversibillity of toxicological changes induced by the tested substance. Satellite groups were restricted to the highest dose level and the control [18]. In this experiment, the highest dose of the tamarind pulp water extract was 1000 mg/kg bw per day. Based on OECD 2009, a limit of 1000 mg/kg bw must be applied, except when human exposure indicates the need for a higher dose level [18].

Rapid body weight loss is a harbinger of ill health or death. Rapid body weight loss could be due to decreased feeding and/or water consumption, disease or specific toxic effects [19]_._ In this study, there were no significant body weight changes in male and female rats of the test group compared to the control group. However, there was a significant increase in body weight in the satellite 1000 mg/kg bw group compared to the satellite control group, one month after tamarind pulp water extract administration was terminated. The reason for the increased body weight of the satellite 1000 mg/kg bw group is still uncertain. The weight gain of the satellite 1000 mg/kg bw group in this current study was still in a normal range [20]. A possible explanation is as follows: tamarind pulp water extract has an effect of inhibition of weight gain [16]. When the administration of tamarind pulp water extract was halted, the body weight of the rats could increase as in the normal condition.

Previous research showed that tamarind pulp water extract (75 mg/kg bw and 225 mg/kg bw) inhibits weight gain in high-carbohydrate diet rats [16] and high fat–induced obese rats [3]. However, this research showed no significant body weight change. It was probably caused by different animal models. In this research, normal rats were used instead of obese-induced rats.

Hematological and blood biochemical parameters are important indicators of general health and toxicity and were assessed at the termination of the sub-chronic and chronic toxicity studies [19]. To investigate the effect of long-term administration of tamarind pulp water extract on the hematological profile, hematological parameters such as hematocrit, red blood cell counts, hemoglobin, platelet counts, white blood cell counts, MCV, MCH and MCHC were evaluated. Blood consists of plasma and formed ellements (cell and cell fragments). Hematocrit or the packed cell volume (PCV) is the percentage of the whole blood volume contributed by formed elements. Red blood cells are the most abudant blood cells. Red blood cells give the whole blood a deep red color because of the red pigment hemoglobin. Hemoglobin is responsible for the cells’ ability to transport oxygen and carbon dioxide. If the hematocrit is low or the Hb content of the red blood cells (RBCs) is reduced, it causes an anemia. Anemia interferes in oxygen delivery to the peripheral tissue. White blood cells or leukocytes defend the body agaist pathogen invasion, and remove toxin, wastes, and abnormal and damaged cells. Platelets are major participants in a vascular clotting system [21]. In this study, there was no significant difference in some hematological parameters such as red blood cell counts, white blood cell counts, hemoglobin, MCH, MCHC, MCV, hematocrit, and platelet counts caused by long-term administration of tamarind pulp water extract. In a previous study, a sub-chronic toxicity study of tamarind pulp water extract showed similar hematological results. The sub-chronic toxicity study (28 days) showed no significant differences in hematological parameters such as the PCV, white blood cell counts, lyphocytes, and monocytes of the extract-administered group as compared to the control [7], although there was a significant increase in neutrofil counts (1800 mg/kg bw and 2700 mg/kg bw) and a significant decrease in eosinofil counts (2700 mg/kg bw and 3600 mg/kg bw) [7]. However, the genotoxicity study of tamarind pulp water extract showed that tamarind pulp extract did not induce the supression of bone marrow cell proliferation [22].

At the end of the toxicity study, the terminal body weights and organ weights were collected. Organ weight is expressed as relative to the body weigth to eliminate the influence of normal variation in animal growth on the interpretation of organ weight data [19]. Organ weight changes can be a sensitive indicator of the target organ toxicity [23]. After six months of administration of tamarind pulp water extract, there were significant differences (*p* < 0.05) in the relative spleen weights of female rats of the 200 mg/kg bw group compared to the control group (0.21 ± 0.03 vs 0.24 ± 0.06). The functions of spleen are removing abnormal blood cells and other blood components by phagocytosis, storing iron, and initiating an immune response to antigens circulating in the blood [21]. The relative spleen weight change should be interpreted in conjunction with histopatologic findings because of the inherent variability in the lymphoid organ weight [24]. Further histopathological evaluation of the spleen should be performed to confirmed the finding in this current study. The relative kidney weights of male rats in the 1000 mg/kg bw group were increased (*p* < 0.05) (0.70 ± 0.07 vs. 0.63 ± 0.05). Changes in the kidney weight may reflect renal toxicity, tubular hyperthropy or chronic progressive nephropaty [24]. Serum urea and creatinine levels in the 1000 mg/kg bw group were not significantly changed. Therefore, the safety of high doses of tamarind pulp water extract on kidneys should be considered.

A clinical chemistry test provides information about hepatocellular function, renal function, and carbohydrate, lipid and protein metabolism. The most frequently used enzymes to asses hepatocellular injury are ALT and aspartate AST. ALT is the most useful enzyme for the detection of hepatocellular injury. Significant elevation of serum ALT activity indicates the release of ALT by hepatocytes. Primarily ALT is cytosolic and its concentration within the cell is up to 10,000 times greater than in the serum. ALT may enter the serum in any condition that sufficiently alters the cell membrane integrity. The urea and creatinine concentration is the most common test used to evaluate renal function. The serum glucose concentration reflects intestinal glucose absorption, hepatic glucose production, and tissue uptake of glucose [25]. After six months of administration of tamarind pulp water extract, there were no significant changes in the clinical biochemistry profiles such as glucose, cholesterol, triglyceride, AST, ALT, urea and creatinine of the tested group compared to the control group. Long-term use of tamarind pulp water extract did not alter the hepatocellular function, renal function, serum glucose and serum lipid concentration. In a previous study, the sub-chronic toxicity of tamarind pulp water extract showed the same results. The total protein, albumin, serum bilirubin, AST and ALT were not significantly different from the control [7].

## 5. Conclusions

There were no abnormalities in hematology and blood biochemistry parameters caused by long-term tamarind pulp water extract administration. This study showed that long-term use of tamarind pulp water extract was generally safe and well tolerated at the tested dose.

## Figures and Tables

**Table 1:** Hematology profile of male rats treated with tamarind pulp water extract for six months.

Groups	White Blood Cell Counts (×10^3^/mm^3^)	Hemoglobin (g/dL)	MCH (pg/sel)	MCHC (g/dL)	Red Blood Cell Counts (×10^6^/mm^3^)	MCV (fL)	Hematocrit (%)	Platelet (×10^3^/mL)
Control	10.95 ± 5.34	14.89 ± 1.58	18.35 ± 1.87	34.94 ± 3.96	8.19 ± 1.17	52.59 ± 0.94	43.12 ± 6.61	317.10 ± 46.76
Control Satellite	10.02 ± 3.77	14.58 ± 1.40	17.55 ± 3.22	33.45 ± 6.80	8.54 ± 1.58	52.68 ± 1.47	45.15 ± 9.34	294.01 ± 62.19
75 mg/kg bw	8.60 ± 3.25	14.87 ± 1.52	17.26 ± 2.17	32.89 ± 4.31	8.78 ± 1.65	52.50 ± 0.81	46.13 ± 8.92	291.61 ± 37.74
200 mg/kg bw	8.18 ± 3.13	14.70 ± 1.90	18.29 ± 2.37	34.95 ± 5.09	8.16 ± 1.42	52.42 ± 0.97	42.88 ± 7.88	303.86 ± 51.07
1000 mg/kg bw	9.50 ± 4.00	14.62 ± 1.85	18.41 ± 2.42	35.24 ± 4.91	8.11 ± 1.69	52.31 ± 0.79	42.49 ± 9.26	301.56 ± 40.15
Satellite 1000 mg/kg bw	9.24 ± 2.22	14.29 ± 1.80	17.81 ± 1.55	33.94 ± 3.11	8.08 ± 1.20	52.49 ± 0.82	42.42 ± 6.42	301.21 ± 51.82

Note: Data were expressed as mean ± SD (standard deviation) (*n* = 19−20), MCH: mean corpuscular hemoglobin; MCHC: mean corpuscular hemoglobin concentration; MCV: mean corpuscular volume; bw: body weight

**Table 2:** Hematology profile of female rats treated with tamarind pulp water extract for six months.

Groups	White Blood Cell Counts (×10^3^/mm^3^)	Hemoglobin (g/dL)	MCH (pg/sel)	MCHC (g/dL)	Red Blood Cell Counts (×10^6^/mm^3^)	MCV (fL)	Hematocrit (%)	Platelet (×10^3^/mL)
Control	7.55 ± 3.03	14.22 ± 2.42	16.95 ± 3.73	35.01 ± 7.57	8.60 ± 1.63	48.40 ± 0.90	41.68 ± 8.27	302.27 ± 65.16
Satellite Control	7.90 ± 3.52	14.78 ± 1.83	17.09 ± 2.12	35.22 ± 4.77	8.75 ± 1.43	48.59 ± 0.90	42.57 ± 7.30	338.32 ± 51.76
75 mg/kg bw	7.33 ± 1.80	13.10 ± 1.29	15.47 ± 1.98	32.32 ± 4.06	8.48 ± 1.66	48.49 ± 0.74	41.13 ± 8.18	276.20 ± 54.16
200 mg/kg bw	9.50 ± 3.05	14.04 ± 1.94	15.62 ± 2.10	31.98 ± 4.54	9.16 ± 1.90	48.91 ± 0.71	44.85 ± 9.49	296.27 ± 62.40
1000 mg/kg bw	9.00 ± 3.44	13.62 ± 1.51	15.88 ± 2.74	32.59 ± 5.75	8.83 ± 1.80	48.77 ± 0.70	43.10 ± 9.03	294.43 ± 54.71
Satellite 1000 mg/kg bw	7.09 ± 1.98	15.03 ± 1.41	15.26 ± 1.38	33.10 ± 4.40	9.51 ± 1.49	48.73 ± 0.50	46.28 ± 7.53	318.14 ± 44.54

Note: Data were expressed as mean ± SD (*n* = 16-20), MCH: mean corpuscular hemoglobin; MCHC: mean corpuscular hemoglobin concentration; MCV: mean corpuscular volume; bw: body weight.

**Table 3:** Relative organ weights of male rats treated with tamarind pulp water extract for six months (%).

Groups	Liver	Lung	Heart	Spleen	Kidneys	Adrenal	Seminal Vesicle	Testicles
Control	3.09 ± 0.39	0.57 ± 0.07	0.36 ± 0.05	0.18 ± 0.08	0.63 ± 0.05	0.02 ± 0.003	0.44 ± 0.07	0.91 ± 0.10
Satellite Control	3.23 ± 0.43	0.59 ± 0.08	0.35 ± 0.07	0.21 ± 0.08	0.66 ± 0.07	0.02 ± 0.003	0.40 ± 0.10	0.85 ± 0.14
75 mg/kg bw	3.10 ± 0.29	0.54 ± 0.07	0.35 ± 0.05	0.21 ± 0.04	0.65 ± 0.06	0.02 ± 0.004	0.49 ± 0.13	0.91 ± 0.16
200 mg/kg bw	3.06 ± 0.26	0.52 ± 0.08	0.34 ± 0.03	0.19 ± 0.06	0.64 ± 0.05	0.02 ± 0.003	0.48 ± 0.13	0.87 ± 0.15
1000 mg/kg bw	3.18 ± 0.38	0.56 ± 0.09	0.36 ± 0.06	0.20 ± 0.04	0.70 ± 0.07 *	0.02 ± 0.004	0.49 ± 0.10	0.93 ± 0.18
Satelit 1000 mg/kg bw	3.16 ± 0.34	0.55 ± 0.05	0.34 ± 0.04	0.19 ± 0.04	0.63 ± 0.05	0.02 ± 0.003	0.41 ± 0.09	0.78 ± 0.07

Note: Data were expressed as mean ± SD (*n* = 18–20), *: *p* < 0.05 compared to control group.

**Table 4:** Relative organ weights of female rats treated with tamarind pulp water extract for six months (%).

Groups			Liver	Lung	Heart	Spleen	Kidneys	Adrenal	Ovaries	Uterus
Control			3.71 ± 0.67	0.71 ± 0.17	0.38 ± 0.05	0.24 ± 0.06	0.66 ± 0.08	0.03 ± 0.005	0.05 ± 0.02	0.29 ± 0.12
SatelliteControl			3.66 ± 0.72	0.72 ± 0.13	0.35 ± 0.03	0.24 ± 0.05	0.70 ± 0.08	0.03 ± 0.006	0.05 ± 0.01	0.33 ± 0.10
75 mg/kg bw			3.54 ± 0.57	0.69 ± 0.11	0.36 ± 0.03	0.22 ± 0.04	0.64 ± 0.06	0.03 ± 0.004	0.05 ± 0.01	0.23 ± 0.06
200 mg/kg bw			3.40 ± 0.40	0.67 ± 0.11	0.36 ± 0.02	0.21 ± 0.03 *	0.64 ± 0.07	0.03 ± 0.006	0.05 ± 0.02	0.25 ± 0.07
1000 mg/kg bw			3.68 ± 0.56	0.65 ± 0.12	0.37 ± 0.03	0.22 ± 0.03	0.66 ± 0.08	0.03 ± 0.005	0.06 ± 0.01	0.26 ± 0.07
Satellite 1000mg/kg bw			3.67 ± 0.46	0.65 ± 0.09	0.35 ± 0.03	0.23 ± 0.02	0.66 ± 0.06	0.03 ± 0.004	0.05 ± 0.03	0.27 ± 0.09

Note: Data were expressed as mean ± SD (*n* = 16–20), *: *p* < 0.05 compared to control group.

**Table 5:** Clinical biochemistry profile of male rats treated with tamarind pulp water extract for six months.

Groups	Glucose (mg/dL)	Cholesterol (mg/dL)	Triglyceride (mg/dL)	AST (U/L)	ALT (U/L)	Urea (mg/dL)	Creatinine (mg/dL)
Control	110.10 ± 20.18	43.90 ± 10.04	90.36 ± 21.65	145.88 ± 34.98	56.07 ± 11.57	43.14 ± 6.34	0.65 ± 0.16
Satellite Control	97.79 ± 20.10	43.85 ± 9.62	102.60 ± 37.96	139.11 ± 20.90	58.10 ± 8.77	40.41 ± 7.12	0.69 ± 0.15
75 mg/kg bw	121.55 ± 29.42	42.80 ± 9.01	101.85 ± 30.09	138.48 ± 27.89	52.85 ± 10.62	41.13 ± 11.36	0.68 ± 0.14
200 mg/kg bw	112.69 ± 17.51	41.68 ± 12.46	106.58 ± 34.18	132.84 ± 22.04	51.65 ± 10.52	41.12 ± 6.23	0.62 ± 0.08
1000 mg/kg bw	116.66 ± 24.87	47.35 ± 29.17	109.75 ± 55.16	135.99 ± 22.85	49.14 ± 13.11	44.54 ± 7.53	0.69 ± 0.14
Satellite 1000 mg/kg bw	99.37 ± 18.25	41.35 ± 11.13	110.75 ± 35.67	142.68 ± 23.60	59.42 ± 11.10	41.97 ± 6.87	0.63 ± 0.11

Note: Data were expressed as mean ± SD (*n* = 18–20), AST: Aspartate Amino Transferase; ALT: Alanine Amino Transferase.

**Table 6:** Clinical biochemistry profile of female rats with tamarind pulp water extract for six months.

Groups	Glucose (mg/dL)	Cholesterol (mg/dL)	Triglyceride (mg/dL)	AST (U/L)	ALT (U/L)	Urea (mg/dL)	Creatinine (mg/dL)
Control	130.31 ± 43.46	50.62 ± 12.58	134.94 ± 45.21	119.26 ± 39.44	48.48 ± 13.32	43.18 ± 7.91	0.68 ± 0.13
Satellite Control	122.80 ± 54.49	55.90 ± 11.74	134.40 ± 34.51	125.74 ± 33.66	53.18 ± 14.41	42.84 ± 9.05	0.72 ± 0.08
75 mg/kg bw	138.95 ± 46.94	52.60 ± 14.69	122.20 ± 36.07	133.87 ± 42.65	53.46 ± 12.75	44.60 ± 5.94	0.64 ± 0.10
200 mg/kg bw	125.30 ± 30.41	52.15 ± 13.43	116.70 ± 37.32	144.93 ± 60.60	53.71 ± 15.26	45.86 ± 6.70	0.64 ± 0.11
1000 mg/kg bw	116.79 ± 33.95	49.42 ± 12.69	126.58 ± 34.85	125.57 ± 40.65	48.57 ± 13.41	43.75 ± 5.93	0.63 ± 0.16
Satellite 1000 mg/kg bw	113.92 ± 50.75	55.70 ± 9.76	129.80 ± 32.68	119.83 ± 22.89	51.90 ± 12.10	42.70 ± 8.91	0.67 ± 0.06

Note: Data were expressed as mean ± SD (*n* = 16–20), AST: Aspartate Amino Transferase; ALT: Alanine Amino Transferase.
